# Persistent panmixia despite extreme habitat loss and population decline in the threatened tricolored blackbird (*Agelaius tricolor*)

**DOI:** 10.1111/eva.13147

**Published:** 2020-10-31

**Authors:** Kelly Barr, Annabel C. Beichman, Pooneh Kalhori, Jasmine Rajbhandary, Rachael A. Bay, Kristen Ruegg, Thomas B. Smith

**Affiliations:** ^1^ Center for Tropical Research Institute of the Environment and Sustainability University of California, Los Angeles Los Angeles CA USA; ^2^ Department of Ecology and Evolutionary Biology University of California, Los Angeles Los Angeles CA USA; ^3^ Department of Evolution and Ecology University of California, Davis Davis CA USA; ^4^ Department of Biology Colorado State University Fort Collins CO USA

**Keywords:** conservation genetics, demographic modeling, effective population size, gene flow, genomics, habitat loss, threatened species

## Abstract

Habitat loss and alteration has driven many species into decline, often to the point of requiring protection and intervention to avert extinction. Genomic data provide the opportunity to inform conservation and recovery efforts with details about vital evolutionary processes with a resolution far beyond that of traditional genetic approaches. The tricolored blackbird (*Agelaius tricolor*) has suffered severe losses during the previous century largely due to anthropogenic impacts on their habitat. Using a dataset composed of a whole genome paired with reduced representation libraries (RAD‐Seq) from samples collected across the species’ range, we find evidence for panmixia using multiple methods, including PCA (no geographic clustering), admixture analyses (ADMIXTURE and TESS conclude K = 1), and comparisons of genetic differentiation (average FST = 0.029). Demographic modeling approaches recovered an ancient decline that had a strong impact on genetic diversity but did not detect any effect from the known recent decline. We also did not detect any evidence for selection, and hence adaptive variation, at any site, either geographic or genomic. These results indicate that species continues to have high vagility across its range despite population decline and habitat loss and should be managed as a single unit.

## 1 INTRODUCTION

Rising anthropogenic pressures over the past century have created a global biodiversity crisis (Ceballos et al., 2015). Countless species have experienced significant population decline due to habitat reduction and alteration in the course of human activities, and many are now threatened with extinction (Zalasiewicz et al., 2011). Efforts to slow and reverse these trends are often limited by a deficiency of information regarding the evolutionary processes that dictate long‐term species survival (Smith & Bernatchez, 2008). Historically, attempts to fill this information gap employed genetic markers with the capacity for evaluating only coarse genetic patterns (e.g., microsatellites or mitochondrial DNA sequences). A primary objective of these approaches, for instance, was the identification of evolutionarily significant units (ESUs) that may encompass unique and possibly adaptive variation and hence warrant targeted protection (Moritz, 1994; Ryder, 1986). Given the increasing accessibility of genome‐wide data, we can now move toward more precise evaluations of evolutionary processes by directly assessing adaptive variation (Bay et al., 2018; Funk et al., 2012; Ruegg et al., 2018), analyzing fine‐scale gene flow patterns and hierarchical genetic structure (Hendricks et al., 2017; Ruegg et al., 2014; Younger et al., 2017), and estimating recent and historical demographic trends (Beichman et al., 2018, 2019; Oh et al., 2019).

One such species experiencing severe impacts in the course of anthropogenic activities is the tricolored blackbird (tricoloreds; *Agelaius tricolor*), a colonial songbird that is near endemic to California (Beedy et al., 2018). Tricoloreds are now listed as threatened at the state level after declining by an estimated 63% from 1935 to 1975 (Graves et al., 2013) and another 34% from 2007 to 2016 (Robinson et al., 2018). These losses are primarily due to the destruction of the species’ historically preferred habitats for nesting, wetlands, and foraging, grasslands, by extensive agricultural and urban development (Beedy et al., 2018). As a consequence, whereas 93% of surveyed colonies nested in wetlands in the 1930s (Neff, 1937), tricolored colonies today use a broad range of nesting substrates, often including croplands and invasive species (Meese, 2017). Led by a multiagency collaboration of public and private interests (The Tricolored Blackbird Working Group; Kester, 2007), substantial time and financial resources have been committed toward their conservation and recovery over the past two decades. The lone genetic study guiding these efforts, Berg et al. (2010), reported no differentiation and varying levels of genetic diversity among colonies using a small suite of microsatellites and mitochondrial sequences. The limitations of these data leave many questions about range‐wide genetic connectivity and the impacts of population decline on the overall genetic diversity in the species.

Here, we offer a comprehensive examination of current levels of gene flow and genetic diversity in the tricolored blackbird using genome‐wide data. We sample numerous colonies breeding at the range periphery that were not covered by Berg et al (2010) and where the earliest impacts of declining population sizes and restricted gene flow is expected. Using multiple demographic modeling approaches to distinguish between recent and historical events, we assess genetic diversity at multiple temporal scales. Finally, we investigate evidence for local adaptation using outlier and genotype–environment association (GEA) analyses. Our primary objectives are to (a) assess gene flow and genetic diversity, both neutral and adaptive (the latter being the product of local environmental selection), (a) estimate current and long‐term effective population sizes (Ne), and (c) provide management recommendations based upon our results.

## 2 MATERIALS AND METHODS

### 2.1 Genetic sampling

**FIGURE 1:**
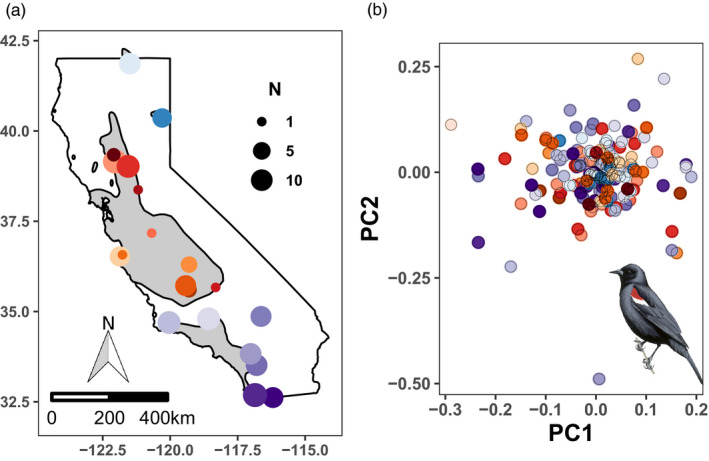
(a) Tricolored blackbird range (gray) and locations of breeding colonies where samples were collected (*N* = 153). Additional details about samples and collection are available in Table S1. (b) Plot of principal components analysis (PCA) of 153 tricoloreds genotyped at 70,933 single nucleotide polymorphisms (SNPs). Each point is an individual colored by general sample location indicated in (a). Clear mixing of genotypes and very low loadings (both <1%) are consistent with panmixia across the breeding range. Warm colors represent colonies sampled in the range core and cool colors are peripheral or southern California

We obtained tissue samples from breeding tricolored colonies throughout their range (Figure 1a). Detailed information about sample sites, numbers of individuals, tissue types, and sources are provided in Table S1. From tissue samples, we purified DNA using DNeasy Blood and Tissue Kits (Qiagen) and assessed extract quantity using a Qubit (Thermofisher) and quality with an agarose gel. We collected genetic data through two means: (a) whole genome sequencing with deep coverage (*n* = 1) and, (b) restriction‐site associated sequencing (RAD‐Seq; *n* = 329).

### 2.2 Genome sequencing

We prepared DNA for whole genome sequencing using the Illumina TruSeq DNA PCR‐Free LT kit (Illumina). After fragmenting 1 µg of DNA to 400 bp using a Diagenode sonicator and cleaning with magnetic beads at a ratio of 105 µl of beads/79 µl of water to select for >400 bp fragments, bioanalyzer traces were collected by the University of California, Los Angeles GenoSeq Core to verify library quality. We sequenced a final library with fragments averaging ~500 bp using a 250 bp paired‐end run on an Illumina HiSeq2500 at the University of California QB3 Vincent J. Coates Genomics Sequencing Laboratory. Scaffolds were assembled from resulting sequence data via the Discovar DeNovo assembler (Broad Institute), and those <5,000 bp were removed. We used BUSCO (Simão et al., 2015) to estimate genome completeness by searching for single copy orthologs common to all species in the class Aves.

### 2.3 Variant discovery

We developed genomic libraries using bestRAD (Ali et al., 2016). For these, DNA was digested using the SbfI restriction enzyme (New England Biolabs, NEB), cleaned using 1X Agencourt AMPure XP beads (Beckman Coulter), ligated with biotinylated adaptors, and sheared to 400bp fragments with a Bioruptor NGS sonicator (Diogenode). We filtered out nonligated fragments using magnetic beads (Dynabeads M‐280; Life Technologies). Blunt ends were repaired and ligated with adaptors via the Illumina NEBNext Ultra DNA Library Prep Kit (NEB), and 500 bp fragments were selected with AMPure beads. PCR‐enrichment was tested using 5 µl of library with a maximum of 15 cycles. Based upon product brightness on an agarose gel, 15 µl of library was then amplified for an appropriate number of cycles, cleaned with AMPure beads, and verified via bioanalyzer traces at the UCLA Technology Center for Genomics and Bioinformatics.

We sequenced RAD‐Seq libraries over four lanes of 100bp paired‐end reads on an Illumina HiSeq2500 at the UC‐Davis DNA Technologies Core, and used the “process_radtags” function in STACKS (Catchen et al., 2013) to demultiplex, filter, trim adapters, and remove low‐quality reads. PCR duplicates were removed using the “clone_filter” function. We mapped reads to the genome assembly with bowtie2 (Langmead & Salzberg, 2012) and identified single nucleotide polymorphisms (SNPs) using the Haplotype Caller module in the Genome Analysis Toolkit (McKenna et al., 2010). We removed low‐quality variants (genotype quality <30, depth <8, minor allele frequency <0.01), indels, and nonbiallelic SNPs with vcftools (Danecek et al., 2011). To determine filtering levels for missing data, we visualized and assessed missingness using the R package “genoscapeRtools” (Anderson, 2019).

### 2.4 Population structure

Population structure is in part a consequence of recent gene flow, and hence is indicative both of a species’ natural and recently developed changes in movement and dispersal patterns. Since closely related individuals can bias signatures of population structure and genetic diversity, we used KING (Manichaikul et al., 2010) to estimate kinship and removed individuals from pairs detected to have first‐order relationships (kinship > 0.177). We conducted principal components analyses (PCA) with the R package SNPRelate (Zheng et al., 2012) and sequentially removed visual outliers. Heterozygosity, both observed (*H*_O_) and expected (*H*_E_), of detected variants and the inbreeding coefficient (*F*_IS_) were estimated with the STACKS POPULATIONS module. We calculated global Tajima's D that is bias‐corrected for missing data and tested for significance with 1,000 simulations in the R package “r2vcftools” (Pope, 2019). Using ADMIXTURE (Alexander et al., 2009) and the spatially explicit Bayesian clustering algorithm TESS (Caye et al., 2016), we estimated the number of genetic clusters in the dataset and assessed individual‐level admixture. We calculated pairwise *F*_ST_ among sample groups (with *N* ≥ 3) using the POPULATIONS module and tested for isolation by distance (IBD) with a Mantel test.

### 2.5 Historical demography and effective population size

While tricoloreds experienced a sharp decline through the 20th century, it is possible that older events also impacted genome‐wide diversity patterns. We examined the species’ demographic history using multiple approaches to understand the impacts of population declines on genetic diversity. These include inferences from the folded site frequency spectrum (SFS), via *∂a∂i* (Gutenkunst et al., 2009) and fastsimcoal2 (Excoffier et al., 2013), and scenario tests using approximate Bayesian computation (ABC) as implemented in DIY‐ABC v2.0 (Cornuet et al., 2014). We also estimated Ne for the current generation based upon linkage disequilibrium (Waples & Do, 2010) using the program NEESTIMATOR v2 (Do et al., 2014).

**FIGURE 3:**
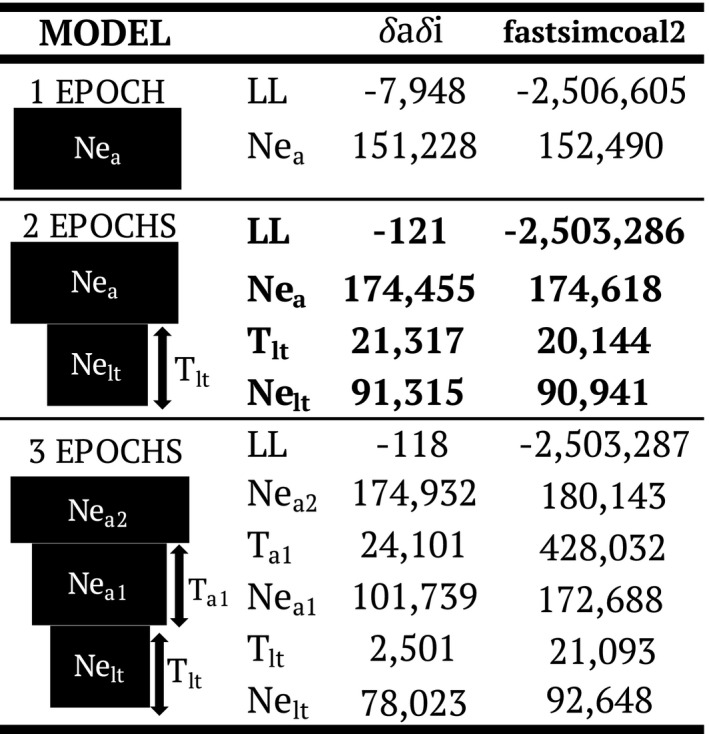
Log‐likelihoods (LL) and parameter estimations (including Time, T, in generations and effective population size, Ne) for the three demographic models examined using *∂a∂i* and fastsimcoal2. The “2 epoch” model was resolved to be best fitting as adding an additional change in Ne had a marginal impact on the LL. NB: The LLs are calculated differently between these two analytical frameworks and hence are not comparable

For both *∂a∂i* and fastsimcoal2, we used an SFS generated via a modification of easySFS (https://github.com/isaacovercast/easySFS) to smooth over missing data and maximize the total number of SNPs using a hypergeometric projection of a dataset filtered to remove loci with >75% heterozygosity. Focusing on historical population size changes for a single population (see results), we compared multiple demographic models: a nested “one epoch” model with no size changes, a “two epoch” model with a single size change, and a “three epoch” model with two size changes (Figure 3). We assumed a mutation rate (μ) of 4.6 × 10–9 (Smeds et al., 2016), generation time (g) of 2 years for both *∂a∂i* and fastsimcoal2, and a sequence length (*L*) of 60,429,389 bps. This *L* is based on sites that had at least 190X coverage (10X * the number of individuals used in the SFS projection) across a merged BAM file composed of the individuals that passed quality filters with and with no close relatives or PCA outliers (see results). Meanwhile, g is based upon a robust estimation from another Passerine (Brommer et al., 2004), and the known ages of first breeding of one year for female and two years for male tricoloreds (Beedy et al., 2018).

In *∂a∂i*, demographic parameters for each model are determined by solving an approximation to the diffusion equation (Gutenkunst et al., 2009). We set extrapolation grid points for simulations as the haploid sample size of the projected SFS and that plus 5, 15, and 25. Using 50 independent replicates, we carried out inferences with permuted starting parameter values and assessed the fit of the expected SFS under the inferred model parameters to the empirical SFS using a multinomial log‐likelihood. For each model, the maximum likelihood estimate (MLE) for the set of 50 runs was selected. The best‐fit θ (population scaled mutation rate) for each replicate's parameters was inferred using *∂a∂i*, and scaled by *L* and μ to calculate the ancestral size in diploids (Ne*_anc_*):

$${\mathrm{Ne}}_{\mathrm{anc}}=\frac{\theta }{4\mu L}$$

Population sizes inferred in *∂a∂i* were then scaled by Ne*_anc_*, and times were scaled by 2*Ne_anc_*g. After determining the best‐fit model, we used a grid‐search approach to refine the bounds of our inferred parameters. We examined a 100x100 grid of values of *nu* (contraction size relative to Ne_anc_) and *T* (contraction duration in terms of 2*Ne_anc_*g) spaced evenly along a log_10_ scale. We obtained the expected SFS for each of the 10,000 parameter pairs using *∂a∂i* and calculated the multinomial log‐likelihood. We then plotted the delta log‐likelihood between each parameter pair and the MLE as a heatmap.

Fastsimcoal2 offers an alternative analytical framework for using the SFS for demographic inferences using coalescent simulations. For each of the previously described demographic models, we ran 100,000 coalescent simulations and used 50 Expected/Conditional Maximization (ECM) parameter‐optimization cycles to estimate the expected SFS for each set of parameters.

We conducted ABC analyses in a sequential, hypothesis‐testing manner with at least 100,000 simulations of each scenario (Figure S6). First, we compared contraction and expansion scenarios, then single versus multiple contractions, and finally, we compared scenarios with a single contraction at four‐time frames: recent (Ta = 0–99 g ago), older (Ta = 100–999 g ago), historical (Ta = 1,000–9,999 g ago), and deeply historical (Ta = 10,000–99,999 g ago). For computational efficiency, we used 1,000 randomly selected loci from the real dataset of 153 individuals and simulated the same number through the scenarios. Using proportion of monomorphic loci, Nei’s (1987) mean gene diversity, variance of gene diversity across polymorphic loci, and mean gene diversity across all loci as summary statistics, we assessed scenario accuracy with a PCA and estimated posterior probabilities of scenarios using both direct and logistic regression approaches. Sampling priors used for simulations are provided in Figure S6.

Estimations of contemporary, short‐term Ne using the LD method are downwardly biased by the presence of overlapping generations (Waples et al., 2014). To limit this effect, we focused estimations on adults sampled in 2017 and 2018, removing nestlings and samples collected in 2002 and 2008. We considered the 132 remaining samples to be a single population based upon the lack of genetic structure (see results). For these samples, we used PLINK2.0 (Chang et al., 2015) to prune varying numbers of SNPs to assess the effect of using reduced representation of the genome on calculations of Ne and report results with a minimum allele frequency (MAF) of 0.01.

### 2.6 Testing for selection

The presence of adaptive variants would be critical information for conservation planning. We used both outlier analyses and genotype–environment association (GEA) methods to detect loci potentially under selection. For the former, we used PCAdapt (Luu et al., 2017) to jointly estimate genetic structure and assess significantly differentiated loci. We used the R package “qvalue” (Storey et al., 2019) to adjust p‐values using the Benjamini–Hochberg correction (Benjamini & Hochberg, 1995). GEAs were assessed using both redundancy analyses (RDA; Forester et al., 2018) and a machine learning approach (gradient forests; Breiman, 2001). For RDA, we used the R package “vegan” (Oksanen et al., 2019) to conduct a permutation test for constrained correspondence for significance testing. Our gradient forest approach detects areas of genotypic transitions associated with environmental conditions. For this, we used the R package “gradientForest” (Ellis et al., 2012) using the following parameters: ntree = 100, nbin = 101, corr.threshold = 0.5. We ran 10 additional gradient forests with randomized environmental variables for confidence testing. Both of these GEAs were based upon 19 climate variables downloaded from WorldClim (Hijmans et al., 2005), the vegetation indices NDVI and NDVIstd for May of 2018 (Carroll et al., 2004), tree cover (Sexton et al., 2013), elevations from the Global Land Cover Facility (www.landcover.org), and surface water measurements (QuickScat; from scp.byu.edu).

## 3 RESULTS

### 3.1 Data quality

We obtained 389 samples from throughout the tricolored's breeding range, including many colonies sampled along range periphery (Figure 1a; Table S1). The genome we assembled for the species is 1.08 Gb in total length across 70,524 scaffolds with an N50 of 103,912 and >100× coverage. Of a total 4,915 known single copy orthologs in Aves, the de novo genome assembly includes 87.2% of these represented completely, 8.3% are fragmented, and 4.5% missing. RAD‐Seq libraries were created for 329 individuals that passed DNA quality standards. After filtering individuals and loci with missing data >10% (*N* = 219) to maximize quantity and quality (Figure S1) and removing close relatives (*N* = 6) and PCA outliers (*N* = 11), a dataset with 153 tricoloreds genotyped at an average of 68,366 SNPs was used for analyses except where indicated. Additional data assessments are provided in Figures S14–S16.

### 3.2 Gene flow and genetic diversity

**FIGURE 2:**
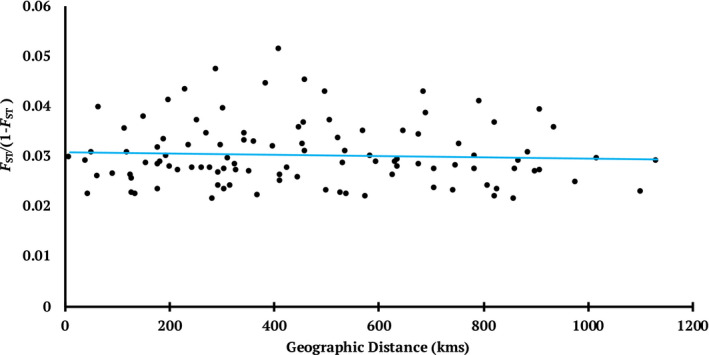
Plot of pairwise genetic distances (normalized *F*_ST_) versus geographic distance (kms) between tricolored blackbird colonies with more than three samples. No significant relationship between differentiation and distances between colonies (Mantel's *r* = −0.11; *p* = .84) indicates high gene flow across the range

We found no evidence for population structure in either PCA (Figure 1b) or clustering analyses—both ADMIXTURE and TESS indicated *K* = 1—suggesting high gene flow across the species’ range. This pattern is supported by similar levels of heterozygosity (*H*_O_: 0.23–0.24; *H*_E_: 0.19–0.22) and inbreeding (*F*_IS_: −0.012 to −0.002) across sample sites (Table S1), which also suggests no individual breeding colonies are in genetic isolation. An excess of rare alleles is suggested in a bias‐corrected Tajima's D was significant (*p* < .001) and positive (1.95; CI: 1.929–1.972), which is a pattern usually attributed to a population expansion after a decline. Average pairwise *F*_ST_ between sampled colonies is quite low at 0.029 (0.02–0.049) and there was no correlation with geographic distance (Figure 2, Mantel's r = −0.11, *p* = .84). This along with a lack of significant differentiation anywhere in the examined genome (Figure S2) further indicates that gene flow is ongoing with no restrictions by either habitat fragmentation or distance.

### 3.3 Historical demography and effective population size

All three demographic modeling approaches indicated a strikingly similar pattern, with the strongest event shaping the species’ genetic diversity being a single contraction that is much deeper in the tricolored's evolutionary history than the known 20th‐century decline. Using an SFS composed of 704,884 SNPs (Figures S3 and S4) from a hypergeometric projection of 153 diploid individuals down to 19, both *∂a∂i,* and fastsimcoal2 rejected the single‐epoch (no size change) model in favor of a two‐epoch contraction (Figure 3; *p*‐value based on likelihood ratio test with two degrees of freedom <.00001). Additional size changes did not significantly improve the fit to the data beyond the two‐epoch model (*p*‐value >.05). ABC results were similar. The expansion and two‐contraction scenarios were sequentially rejected with high confidence (Figures S7 and S8). Meanwhile, the deeply historical contraction scenario was the strongest scenario, suggesting a population decline >10,000 generations ago (Figure S9).

Each of the SFS‐based approaches also arrived at similar parameter estimates of the time since the population contraction and Ne, both Ne_anc_ and long‐term (Ne_lt_). Specific parameter estimates for the two‐epoch model from the grid search in *∂a∂i* suggest ~50% population size decline occurred 21,317 (19,541 – 23,018) g/ago from an Ne_anc_ of 174,455 (173,734 – 175,249) to a Ne_lt_ of 91,315 (89,385 – 92,500; parameter ranges are within 5 log‐likelihood units of the MLE; Figure 3 and S5). The parameters inferred using fastsimcoal2 were highly concordant, exhibiting a decline at 20,144 g/ago from an Ne_anc_ of 174,617 to a Ne_lt_ of 90,941.

**FIGURE 4:**
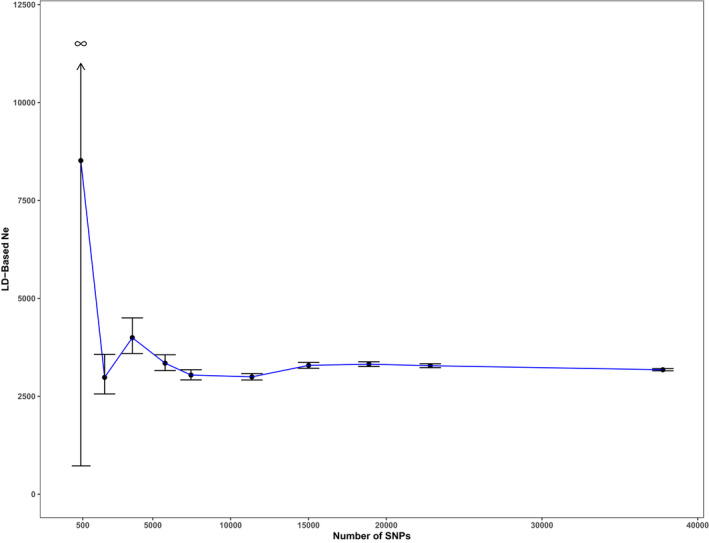
Plot of current effective population size (Ne) estimated from linkage disequilibrium among varying numbers of single nucleotide polymorphisms. The current Ne is ~3,100

While these methods infer a long‐term Ne_lt_ of ~91,000, the LD method suggests current Ne is much lower at ~3,100 (Figure 4). These methods are not directly comparable as SFS‐based approaches are more influenced by ancient events and the LD estimation is the product of recent genetic drift. Further, all methods are influenced by model violations in different ways. SFS‐based inferences, for instance, maybe impacted by incorrect mutation rates, and the LD method may be sensitive to cryptic linkage. For instance, if we examine the impacts on *∂a∂i* results of alternative μ used by previous authors for other birds (Nadachowska‐Brzyska et al., 2015), it is apparent that a lower rate assumed for a domestic pigeon (*Columba livia*; mu = 4.598e−10) results in Ne_lt_ and g since the contraction are 10X as large; meanwhile, a higher rate such as that used for the rhinoceros hornbill (*Buceros rhinoceros*; mu = 6.999e−10) results in estimations that are 50% smaller (Table S3). The μ we choose to use here from Smeds et al. (2016) seems to be the most robustly estimated one available for a fellow songbird.

### 3.4 Testing for selection

There was no evidence for strong selection across the species’ genome. The scree plot of eigenvalues calculated in PCAdapt exhibited no sharp changes in proportion of explained variation, which is the pattern expected when there is no population structure or significant outlier loci (Figure S10). Arbitrarily selecting the first four eigenvalues, no loci were detected as significant outliers after accounting for multiple tests. The GEA correlation in the RDA also found to be insignificant (*p* = .44). Meanwhile, gradient forest analyses revealed both average *r* ^2^ and numbers of correlated SNPs from randomized environmental datasets were higher than those detected from the empirical data (Figure S11), suggesting any correlations detected are spurious and likely variable between runs. Despite a sampling effort aimed to capture a broad range of habitat and environmental conditions experienced by tricoloreds, there were no adaptive variants apparent in our dataset.

## 4 DISCUSSION

### 4.1 Gene flow and genetic diversity

Our data provide multiple lines of evidence indicating that tricoloreds persist as a single, panmictic population. The low genetic differentiation we observed both among breeding colonies and across the genome is the product of high gene flow that encompasses their complete range. Breeding colonies separated by the greatest distances, often with little suitable breeding or foraging habitat in between, are no more differentiated than the closest ones. This is particularly striking given the focus on peripheral, disparate colonies in the sample design. Most of the birds breed in the core of their contemporary range in the Central Valley of California, and colonies at the periphery tend to be comparatively small. If isolated, genetic drift would be expected to rapidly cause differentiation and loss of genetic diversity in these smaller colonies. Here, however, the high genetic connectivity we observed even at the extremes of the breeding range further supports the conclusion that the species is in panmixia.

Our genome‐wide analyses also illustrate higher and more far‐reaching vagility by tricoloreds than previously known, though multiple studies employing bands on thousands of individuals have revealed large‐scale movements over major portions of the breeding range. These include, for instance, across the Central Valley (Beedy et al., 2018; DeHaven et al., 1975; Neff, 1942), along the coast (Wilson et al., 2016), and throughout southern California (Neff, 1942). However, because movements were not observed between the Central Valley and southern California, in particular by Neff (1942), the prevailing notion was that tricoloreds should be considered two separate demes split between these areas. This is illustrated in an equivocal presentation of genetic diversity calculations for these two areas by Berg et al. (2010) even though they observed no significant genetic differentiation. High vagility and panmixia are important conclusions for conservation efforts, as the species as a whole can be considered a single deme.

It is notable that high genetic connectivity persists across the range despite the severe population decline and habitat loss experienced by the species over the past century. This may not be surprising for a vagile, volant species; however, genetic isolation associated with habitat fragmentation over similarly narrow extents was previously reported in other songbirds (Barr et al., 2008, 2015; Lindsay et al., 2008) and species with high dispersal distances would be expected to exhibit the earliest impacts of a barrier to gene flow (Landguth et al., 2010). It is possible that the species’ historical preference for nesting in seasonal wetland habitat, which is inherently ephemeral in western North America, contributes to their capacity for maintaining genetic connectivity despite severe habitat loss. Since wetlands may not develop in the same geographic locations on an annual basis, the species is likely adapted for searching over greater areas for suitable habitat (Cerame et al., 2014). Indeed, the existence of the many colonies in extreme geographic isolation we sampled for this study further suggests a broadly ranging habitat searching behavior by tricoloreds.

### 4.2 Historical demography and effective population size

Considering the documented extreme decline tricoloreds experienced during the past century, it is surprising that the multiple demographic modeling approaches we employed uniformly conclude an ancient contraction ~ 20,000 generations ago has more significant impact on shaping genetic diversity in the species. Similar patterns are observed in other species, such as yellow‐bellied toads (*Bombina variegata*; Cornetti et al., 2016) and orcas (*Orcinus orca*; Moura et al., 2014), both of which experienced known recent declines but demographic modeling illustrates events deeper in evolutionary time are more impactful for shaping long‐term genetic diversity. Though ABC methods are frequently employed for examining recent bottlenecks (e.g., Cammen et al., 2018; Richmond et al., 2013; Xenikoudakis et al., 2015), our dataset may be too coarse for detecting the impacts of the known 20th‐century decline in tricoloreds given its recency and the relatively high remaining population size (2017 Nc = 177,656; Meese, 2017). It is clear from our analyses, though, that an ancient contraction occurred was highly consequential in shaping overall species genetic diversity.

While there are no clear causes of the inferred ancient decline, plausible explanations include climate change or species divergence. During high glacial periods, precipitation was high in western North America (Allen & Anderson, 1993; Oster et al., 2015) resulting in more abundant wetland habitat and likely higher tricolored population sizes. Assuming a generation time of 2 years (Brommer et al., 2004), the decline was older than the Last Glacial Maximum (20,000 y/ago); however there were numerous climatic oscillations between 20,000 and 60,000 year/ago (Petit et al., 1999) that could have resulted in significant increases or decreases in breeding habitat. As for the contraction signal being a recent species divergence, it is notable that the node between tricolored and red‐winged blackbirds (*Agelaius phoeniceus*) is less well resolved than most others in an Icteridae phylogeny (Powell et al., 2014). This apparent incomplete lineage sorting is suggestive of a recent divergence between the species and possibly postdivergence hybridization. We also collected RAD‐Seq libraries for ten red‐wingeds (sampled next to tricolored colonies) to test for the possibility of hybridization but found no evidence for admixture between these closely related species that often share breeding habitat (Figures S12 and S13). TimeTree (Kumar et al., 2017) indicates these two blackbird species diverged long before (>3 Mya; based upon (Barker et al., 2015; Powell et al., 2014)) the estimated time of population contraction detected here (~40,000 years ago). This suggests the ancient cause of population decline is more likely associated with Pleistocene climate change rather than speciation.

Both the long‐term Ne (~90,000) and recent Ne (~3,100) estimated here are surprisingly low given the 400,000 birds reported in a 2008 census (Kelsey, 2008) and early 1900s estimates numbering in the millions. Ne is generally smaller than Nc, and the ratio between these varies between species based upon life history characteristics (Frankham, 1995). Long‐term Ne, for instance, is influenced by population fluctuations over time, with small sizes having the strongest effect (Vucetich et al., 1997). This is relevant to tricoloreds as their populations likely fluctuated throughout its evolutionary history due to interannual variation in both in prey abundance (Meese, 2013) and habitat availability. Another factor that influences long‐term Ne is variance in reproductive success, with high variance reducing the ratio of Ne to Nc (Sugg & Chesser, 1994). Thus, polygyny, which is thought to be relatively high in tricoloreds (Liu, 2014), may also impact Ne in an unpredictable direction that would require additional information about the mating system to ascertain (Liu, 2015). Finally, beyond the aforementioned biological causes, there is also an analytical component that should be considered. Our current N_e_ calculation may be reduced by the presence of overlapping generations (Waples et al., 2014), which is likely in our dataset because adults are long‐lived (~12 years) and a lack of variation in molt and plumage beyond the second year limits age assessments (Beedy et al., 2018).

### 4.3 Evidence for selection

While full whole genome sequences are indisputably better for assessing subtle genetic variation patterns, our results illustrate no major selective sweeps affecting large regions of the tricolored genome. It is notable that such evidence has been reported with less sequencing effort in other species with much larger genomes (e.g., Hohenlohe et al., 2010; White et al., 2013). This lack of evidence for selection may not be surprising given both the low standing genetic variation apparent in our Ne estimates and the recent, widescale shifts in habitat uses from primarily wetlands to highly variable alternative nesting substrates (Beedy et al., 2018; Meese, 2017). Another possible limitation to the development of adaptive diversity is gene swamping (Lenormand, 2002) across the range by dispersers from the Central Valley, where most of the species breeds. The high gene flow we detected would likely preclude the rise of large‐effect alleles around the range periphery, where variance in environmental conditions is highest (Kirkpatrick & Barton, 1997). Additional sequencing effort focused on full genomes would be helpful for analyzing adaptive variation associated with alleles of weaker effect that may develop despite gene flow (Tigano & Friesen, 2016) and examining targeted regions directly relevant to a species’ long‐term viability, such as MHC loci (Agudo et al., 2012).

## 5 CONCLUSIONS AND RELEVANCE TO MANAGEMENT

Our results illustrate the analytical power and additional information gained from reexamining a system only informed by classical genetic markers with a modern genomic approach. Berg et al. (2010) were generally inconclusive about gene flow in the species, reporting at the same time a lack of genetic differentiation but also differences in genetic diversity among sample sites. Our data allow us to conclude that tricoloreds may be managed without concern for gene flow, directed preservation of unique genetic variation, or a focus on recovery of any particular local aggregation anywhere in their range. Genetic diversity, while seemingly low overall, is homogenous across breeding colonies. These results indicate the species as a whole may justifiably be considered a single management unit.

It seems that the tricolored's natural history modulates species‐wide genetic diversity and that the current level is quite a bit lower than one might predict given their recent census population sizes. While we detected no evidence for inbreeding, whether through population‐level estimations of *F*_IS_ (Table S1) or analyses of runs of homozygosity within individuals (data not shown), the current Ne suggests that ongoing genetic monitoring should occur to supplement censuses. Moreover, given the heterozygote excess we observed at all sample sites (Table S1), tricoloreds are likely in “drift debt” (Gilroy et al., 2017) and will experience further erosion of genetic diversity as they settle into mutation‐drift equilibrium.

Our dataset and sample design should be quite powerful for the analyses we report here; however, we cannot entirely discount the possibility that our reduced representation dataset may miss weak or burgeoning genetic differentiation. Future additional sequencing effort aimed at whole genomes would significantly increase our power for detecting weak genetic differentiation or selection, and allow for a finer‐scaled assessment of genetic diversity by estimating genome‐wide heterozygosity. The relative impacts of the ancient and recent contraction events may be further examined through alternative analytical techniques that are less sensitive to departures from model assumptions, such as using identity by descent segments (Browning & Browning, 2015). Finally, museum samples may be used to better understand the impacts of recent population decline on the genetic diversity of the species.

## ANIMAL WELFARE AND PERMIT STATEMENTS

Samples were collected under Tom Smith's Federal Bird Banding Permit, #21901, and Kelly Barr's Scientific Collecting Permit, #SC‐11568, and an MOU with California Fish and Wildlife. Animal handling and sampling protocols were conducted with the approval of UCLA’s Animal Research Committee (ARC), agreement #2017‐073–03.

## CONFLICT OF INTEREST

None declared.

## AUTHOR CONTRIBUTIONS

KRB, ACB, PK, RAB, KR, and TBS helped design the research and write the paper. RAB designed the bioinformatic pipeline for processing raw data, and both assembled and assessed the quality of the de novo genome. KRB and JR conducted fieldwork and laboratory work. ACB and PK ran SFS inferences. KRB completed all remaining analyses.

## Supporting information

Supplementary Material

## Data Availability

All of the genetic data collected for this study are available in public databases. These include the genome assembly in the NCBI Sequence Read Archive, accession SAMN16392922, and vcf files, both filtered and unfiltered, on Dryad, https://doi.org/10.5068/D1DM4H.
