# Supplementary material for: Persistent panmixia despite extreme habitat loss and population decline in the threatened tricolored blackbird (Agelaius tricolor)
Source: Evol Appl. 2020 Oct 31;14(3):674–84. doi: 10.1111/eva.13147 (PMC7980274; doi:10.1111/eva.13147)
Supplement: Supplementary file 1 — Supplementary Material [file EVA-14-674-s001.docx]

SUPPLEMENTARY MATERIALS

Table S1. Sample site details, including locations* (map ID colors correlate with those in Figures 1A and 1B), samples included in most analyses (N), tissue types (if known), and diversity indices. These include observed and expected heterozygosities (H_O_ and H_E_), nucleotide diversity (pi), and the inbreeding coefficient (*F*_IS_).

*Exact coordinates withheld at landowner request.

**Samples are from Berg et al. 2010.

^1^Samples collected using passive mistnets and via a puncture of the brachial vein (Stangel 1986; Sheldon et al. 2008) to obtain blood

^2^Samples were provided by Emily Graves, UC-Davis

^3^Samples were provided by Dr. Kristie Wychoff, Santa Lucia Conservancy

^4^Samples were provided by Jessie Bahm, USDA.

^5^Samples were provided by Dr. Robert Meese


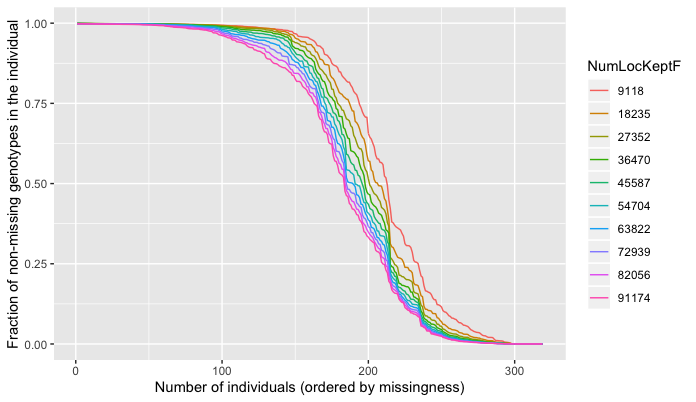


Figure S1. Levels of missingness across varying numbers of individuals. This is produced by the R package ‘genoscapeRtools’ (Anderson 2019).


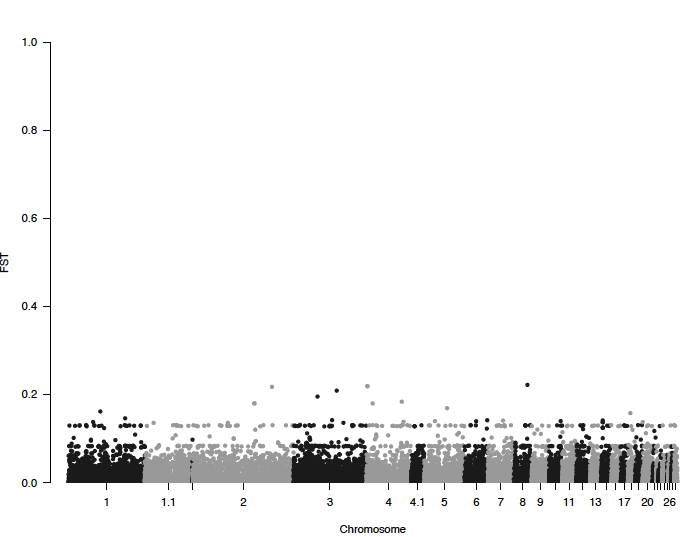


Figure S2. Differentiation (*F*_ST_) across the Tricolored Blackbird genome based upon sites with three or more samples. Low genome-wide *F*_ST_ is indicative of panmixia in the species.

Figure S3. The projection preview from easySFS showing the number of SNPs for each possible projection value in terms of haploid individuals. The number of SNPs is maximized at 38 haploids (19 diploids), yielding 704,884 SNPs, so this value was chosen for the projection.

Figure S4. The projected folded site frequency spectrum, showing the number of SNPs along the y-axis that are present in the sample at each frequency (x-axis). The SFS was projected down from 153 individuals to 19 to maximize the number of SNPs and smooth over missing data.


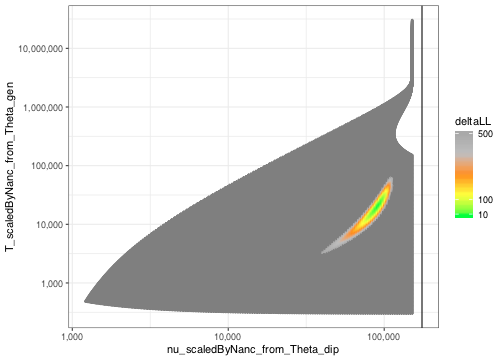

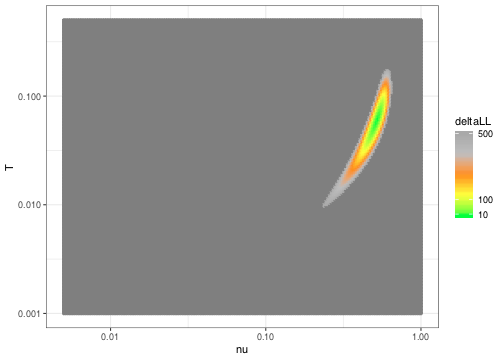


B

A

Figure S5. Heatmaps summarizing results of demographic inference in ∂a∂i*.* For the best fit “2 epoch” model, we examined a 100x100 range of parameters *nu* (contraction size scaled by N_anc_) and T_lt_ (time since contraction scaled to 2*N_anc_ generations), spaced evenly along a log-scale, and colored by the delta log-likelihood (LL) between the expected site frequency spectrum (SFS) for each parameter pair and the maximum likelihood estimate (MLE). A ridge of high-likelihood estimates can be seen in green, with a correlation between contraction size and duration, corresponding to a ~50% contraction in population size ~20,000 generations ago. Long-term effective population is estimated to be ~91,000. (A) presents these results in terms of *nu* (x-axis; contraction size scaled by N_anc_) and T_lt_ (y-axis; time since contraction scaled to 2*N_anc_ generations) and (B) shows the same results converted into units of diploid individuals (x-axis; Ne_lt_) and generations (y-axis; g), based on the best-fit estimate of 𝛳 from ∂a∂i, and scaled using a mutation rate ($\mu)$ of 4.6 x 10-9 (Smeds et al., 2016) and a sequence length (L) of 60,429,389 bps.

Figure S6. Approximate Bayesian Computation (ABC) parameter sampling priors on left and demographic scenarios simulated on right. Most likely scenarios as determined through direct and logistic regression are indicated with an asterisk (*).


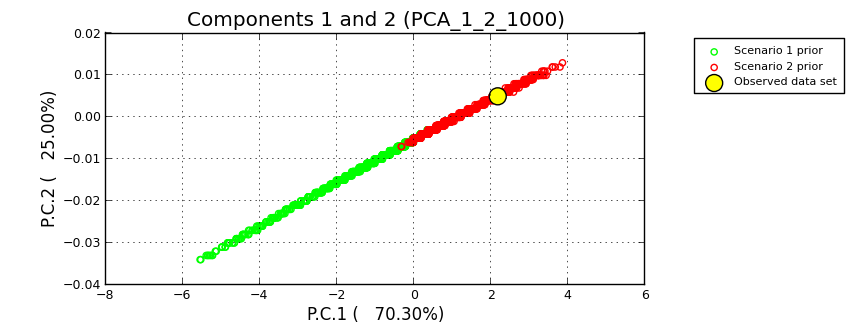

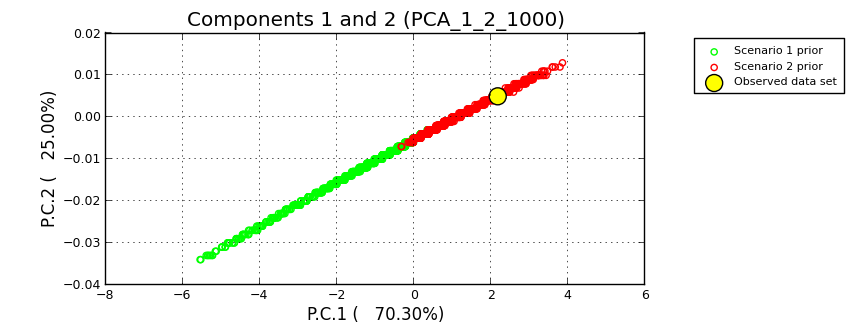

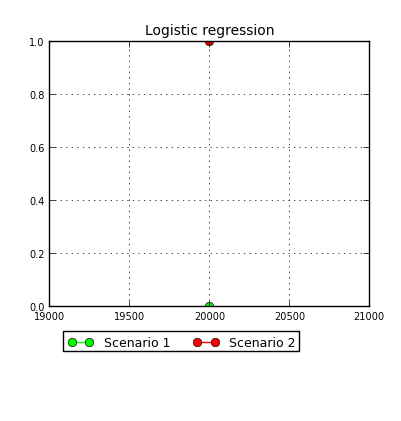


FigureS7. Results of ABC comparison of (1) expansion and (2) contraction scenarios with PCA on left and logistic regression on right. The direct (not presented) and logistic results both suggest a contraction is the most likely scenario.


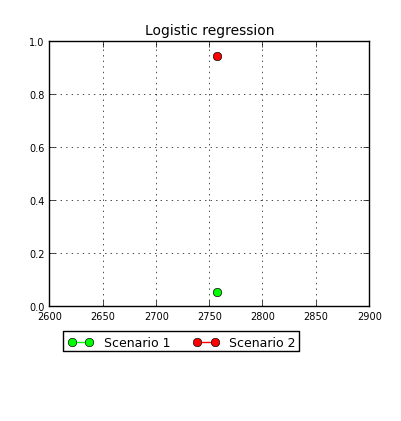

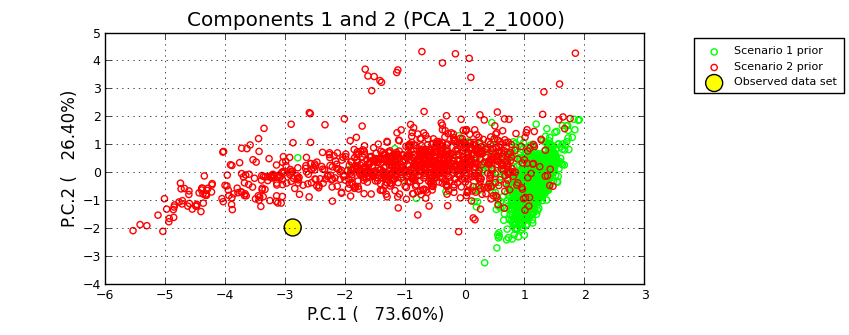

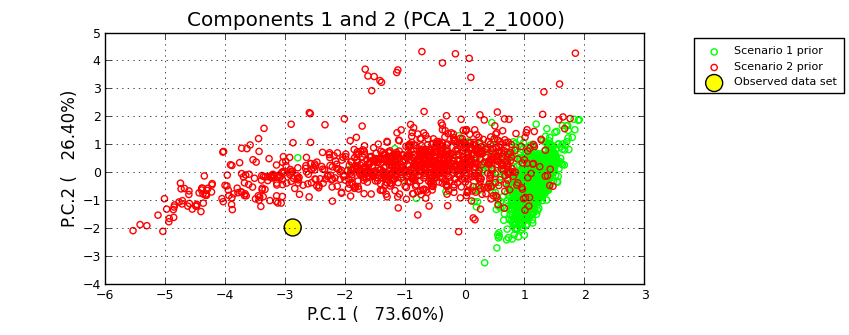


FigureS8. Results of ABC comparison of (1) two contraction and (2) one contraction scenarios with PCA on left and logistic regression on right. The direct (not presented) and logistic results both suggest a single contraction is the most likely scenario.


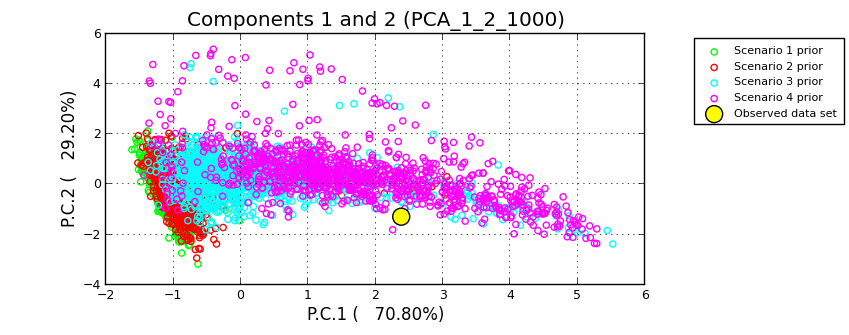

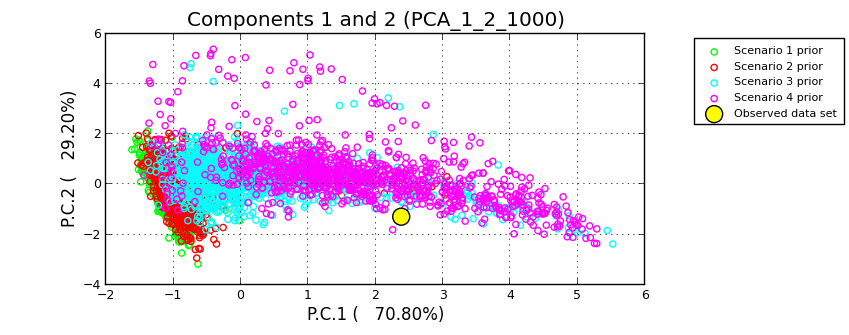

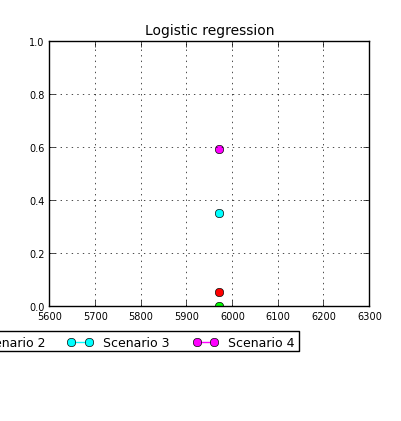


FigureS9. Results of ABC comparison of (1) recent (previous 1-99 generations/ago), (2) older (100 – 999 generations/ago), (3) historical (1000 – 9999 generations/ago), and (4) deeply historical (10000 – 99999 generations/ago) contraction scenarios with PCA on left and logistic regression on right. The direct (not presented) and logistic results both suggest a deeply historical contraction is the most likely scenario.

Figure S10. Scree plot of PCadapt analysis. Miniscule gains in proportion of explained variance as additional PCs are added is consistent with both a lack of genetic structure and no outlier loci.


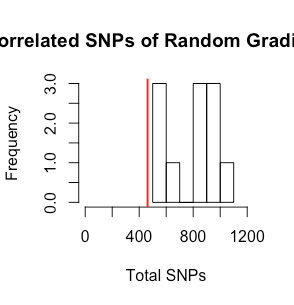

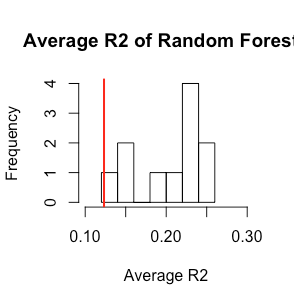


Figure S11. Results of ten randomized gradient forests compared to empirical results. Higher frequencies of total SNPs (left) and average r^2^ (right) indicate the empirical results (red line) are spurious and the gradient forest model in insignificant.


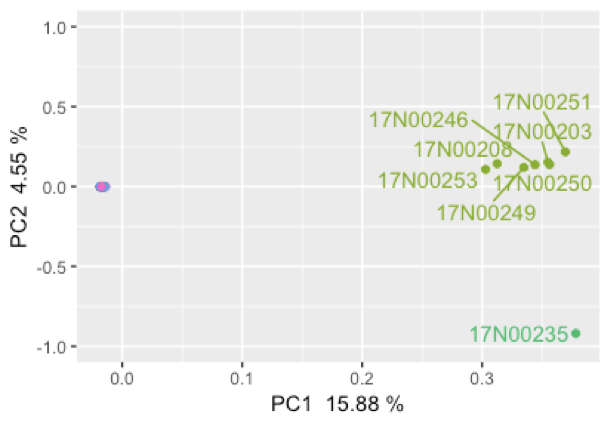


Figure S12. PCA results that include red-winged and tricolored blackbird RAD-Seq data. The former samples are labeled by individual sample names on the right, and all 153 of the latter samples are on the left. The lack of intermediate genotypes between red-wingeds and tricoloreds is evident with the lack of intermediate PCA results. There is also a notable lack of variation within tricoloreds (all lumped together) versus wide variation apparent in the 10 red-winged samples (interspecific variation is captured in PC2).


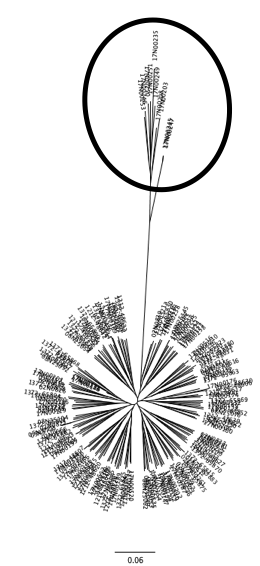
Figure S13. RAXML analysis of Red-winged and Tricolored Blackbirds SNP data. Red-winged samples are circled. As with the PCA in Figure S12, the lack of intermediate results between the 153 Tricoloreds in the big group at the bottom and the 10 Red-wingeds circled is evidence for a lack of intermediate genotypes between the species.

Some Extra Figures

Figure S14. Proportions of expected genotypes (x-axis) and observed (y-axis) as estimated in the R package “whoa” (https://github.com/eriqande/whoa). Loci appearing on the “envelope” of the homozygous genotypes (0, 2) with zero observations and as being totally heterozygous (geno 1) are likely paralogous loci. There are 900 such loci in this dataset.

Figure S15. Depth calculated across loci. We considered loci with depths greater than the mean (38.9X) + one standard deviation (43.6X) to be potential paralogous loci. We found 932 loci that were >82.5X.

Figure S16. PCA after removing potential paralogous loci (N=1375) and those in high LD (N=7819). Sample colors correlate with sample sites in Figures 1 and S1. Outlying samples here are likely second order relatives as estimated in PLINK2.0. The two in the bottom left along PC1 have a kinship of 0.087 and those at top right along PC2 0.073.

Table S2. Diversity statistics after removing paralogous and high LD loci. All statistics are significantly correlated with their corollaries in table S1 (Ho: Pearson’s r = 0.99, p < 0.00001; He: r = 0.99, p < 0.00001; pi: r = 0.98, p < 0.00001; Fis: r = 0.95, p < 0.00001).

Table S3. Illustrating the effects of varying mutation rates on ∂a∂i inferences. The original rate is from Smeds et al. (2016) and the lower and higher rates are from Nadachowska-Brzyska et al. (2015). Parameters are as estimated in ∂a∂i for the two epoch model.
